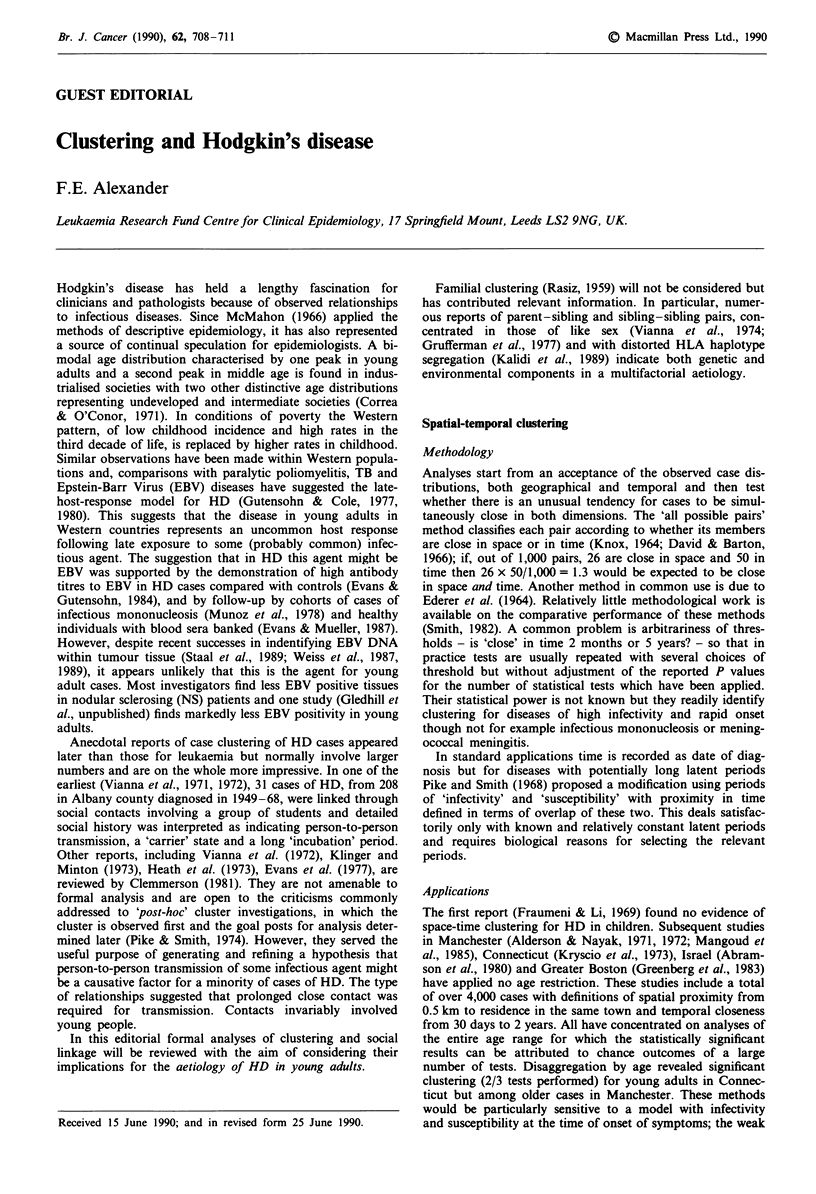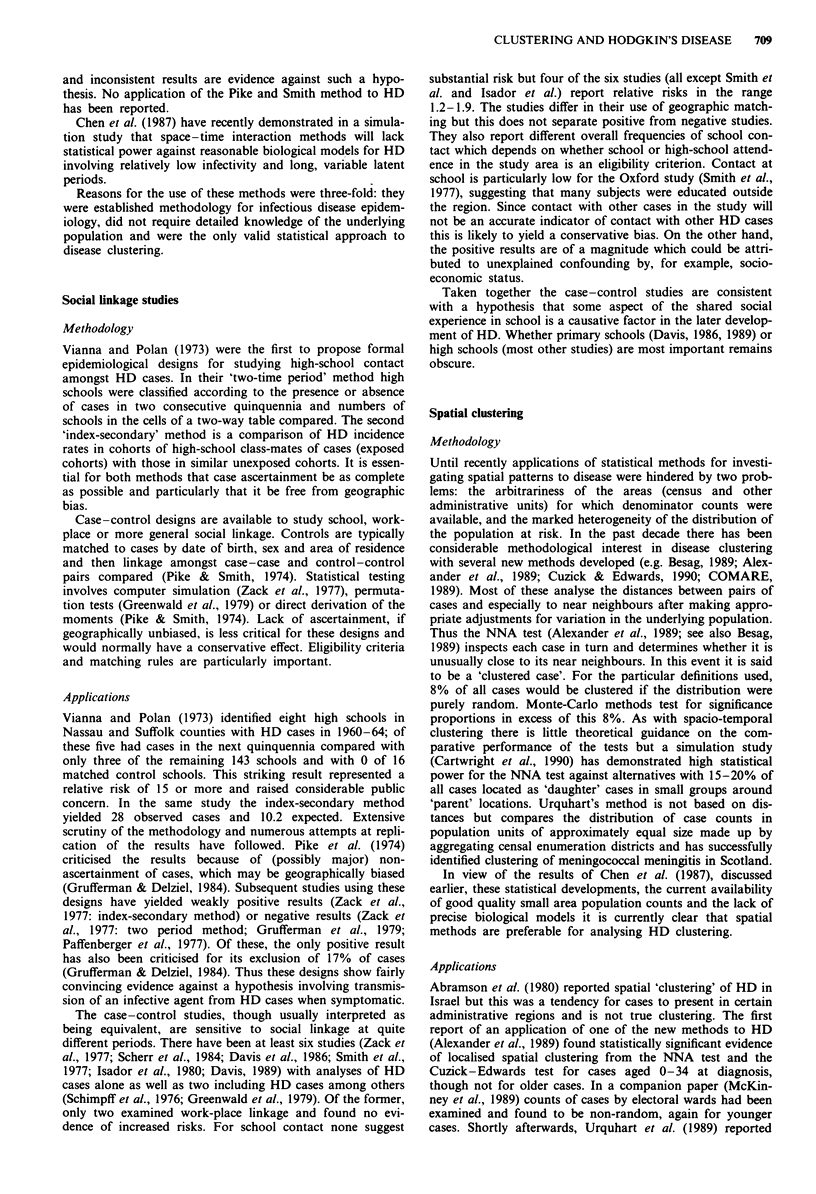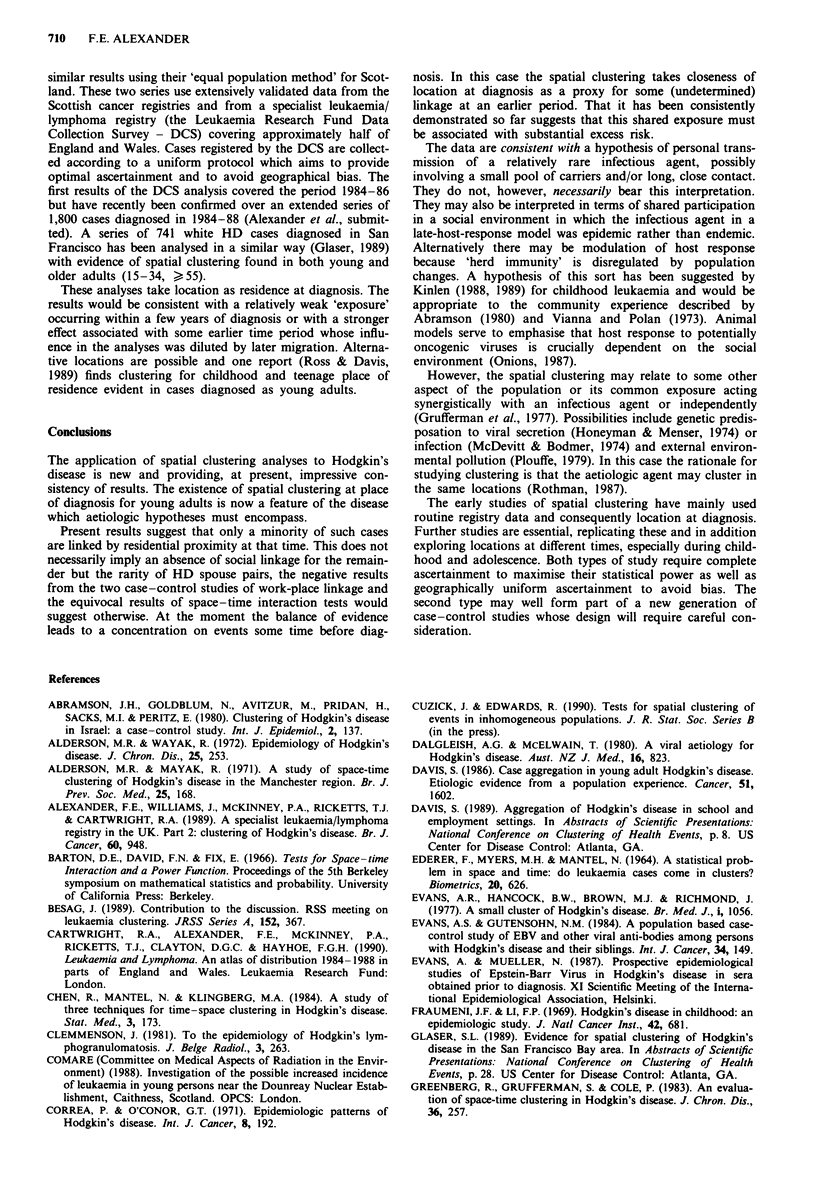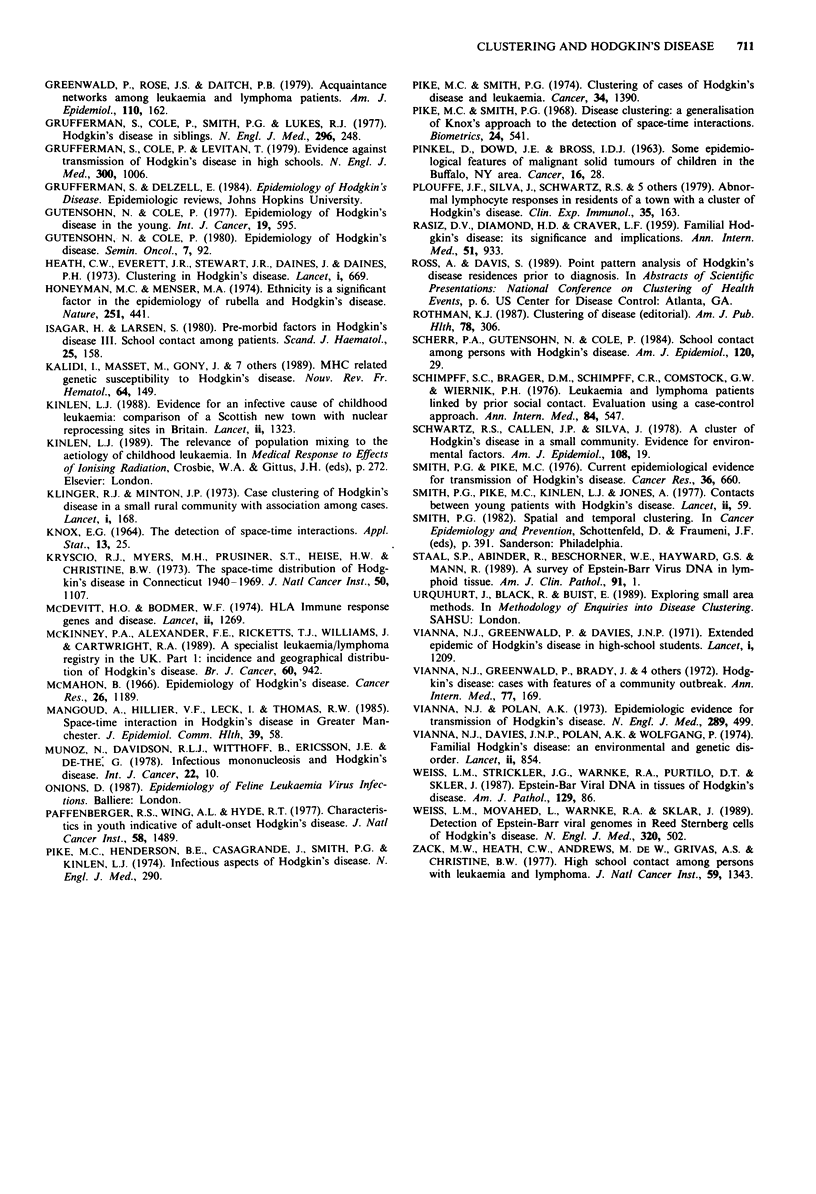# Clustering and Hodgkin's disease.

**DOI:** 10.1038/bjc.1990.364

**Published:** 1990-11

**Authors:** F. E. Alexander


Br. J. Cancer (1990), 62, 708–711 © Macmillan Press Ltd., 1990

### GUEST EDITORIAL

### Clustering and Hodgkin's disease

F.E. Alexander

*Leukaemia Research Fund Centre for Clinical Epidemiology, 17 Springfield Mount, Leeds LS2 9NG, UK.*

Hodgkin's disease has held a lengthy fascination for clinicians and pathologists because of observed relationships to infectious diseases. Since McMahon (1966) applied the methods of descriptive epidemiology, it has also represented a source of continual speculation for epidemiologists. A bimodal age distribution characterised by one peak in young adults and a second peak in middle age is found in industrialised societies with two other distinctive age distributions representing undeveloped and intermediate societies (Correa & O'Conor, 1971). In conditions of poverty the Western pattern, of low childhood incidence and high rates in the third decade of life, is replaced by higher rates in childhood. Similar observations have been made within Western populations and, comparisons with paralytic poliomyelitis, TB and Epstein-Barr Virus (EBV) diseases have suggested the late-host-response model for HD (Gutensohn & Cole, 1977, 1980). This suggests that the disease in young adults in Western countries represents an uncommon host response following late exposure to some (probably common) infectious agent. The suggestion that in HD this agent might be EBV was supported by the demonstration of high antibody titres to EBV in HD cases compared with controls (Evans & Gutensohn, 1984), and by follow-up by cohorts of cases of infectious mononucleosis (Munoz *et al.*, 1978) and healthy individuals with blood sera banked (Evans & Mueller, 1987). However, despite recent successes in indentifying EBV DNA within tumour tissue (Staal *et al.*, 1989; Weiss *et al.*, 1987, 1989), it appears unlikely that this is the agent for young adult cases. Most investigators find less EBV positive tissues in nodular sclerosing (NS) patients and one study (Gledhill *et al.*, unpublished) finds markedly less EBV positivity in young adults.

Anecdotal reports of case clustering of HD cases appeared later than those for leukaemia but normally involve larger numbers and are on the whole more impressive. In one of the earliest (Vianna *et al.*, 1971, 1972), 31 cases of HD, from 208 in Albany county diagnosed in 1949–68, were linked through social contacts involving a group of students and detailed social history was interpreted as indicating person-to-person transmission, a 'carrier' state and a long 'incubation' period. Other reports, including Vianna *et al.* (1972), Klinger and Minton (1973), Heath *et al.* (1973), Evans *et al.* (1977), are reviewed by Clemmerson (1981). They are not amenable to formal analysis and are open to the criticisms commonly addressed to '*post-hoc*' cluster investigations, in which the cluster is observed first and the goal posts for analysis determined later (Pike & Smith, 1974). However, they served the useful purpose of generating and refining a hypothesis that person-to-person transmission of some infectious agent might be a causative factor for a minority of cases of HD. The type of relationships suggested that prolonged close contact was required for transmission. Contacts invariably involved young people.

In this editorial formal analyses of clustering and social linkage will be reviewed with the aim of considering their implications for the *aetiology of HD in young adults*.

Received 15 June 1990; and in revised form 25 June 1990.

Familial clustering (Rasiz, 1959) will not be considered but has contributed relevant information. In particular, numerous reports of parent–sibling and sibling–sibling pairs, concentrated in those of like sex (Vianna *et al.*, 1974; Grufferman *et al.*, 1977) and with distorted HLA haplotype segregation (Kalidi *et al.*, 1989) indicate both genetic and environmental components in a multifactorial aetiology.

### Spatial-temporal clustering

### *Methodology*

Analyses start from an acceptance of the observed case distributions, both geographical and temporal and then test whether there is an unusual tendency for cases to be simultaneously close in both dimensions. The 'all possible pairs' method classifies each pair according to whether its members are close in space or in time (Knox, 1964; David & Barton, 1966); if, out of 1,000 pairs, 26 are close in space and 50 in time then $26 \times 50/1,000 = 1.3$ would be expected to be close in space *and* time. Another method in common use is due to Ederer *et al.* (1964). Relatively little methodological work is available on the comparative performance of these methods (Smith, 1982). A common problem is arbitrariness of thresholds – is 'close' in time 2 months or 5 years? – so that in practice tests are usually repeated with several choices of threshold but without adjustment of the reported $P$ values for the number of statistical tests which have been applied. Their statistical power is not known but they readily identify clustering for diseases of high infectivity and rapid onset though not for example infectious mononucleosis or meningococcal meningitis.

In standard applications time is recorded as date of diagnosis but for diseases with potentially long latent periods Pike and Smith (1968) proposed a modification using periods of 'infectivity' and 'susceptibility' with proximity in time defined in terms of overlap of these two. This deals satisfactorily only with known and relatively constant latent periods and requires biological reasons for selecting the relevant periods.

### *Applications*

The first report (Fraumeni & Li, 1969) found no evidence of space-time clustering for HD in children. Subsequent studies in Manchester (Alderson & Nayak, 1971, 1972; Mangoud *et al.*, 1985), Connecticut (Kryscio *et al.*, 1973), Israel (Abramson *et al.*, 1980) and Greater Boston (Greenberg *et al.*, 1983) have applied no age restriction. These studies include a total of over 4,000 cases with definitions of spatial proximity from 0.5 km to residence in the same town and temporal closeness from 30 days to 2 years. All have concentrated on analyses of the entire age range for which the statistically significant results can be attributed to chance outcomes of a large number of tests. Disaggregation by age revealed significant clustering (2/3 tests performed) for young adults in Connecticut but among older cases in Manchester. These methods would be particularly sensitive to a model with infectivity and susceptibility at the time of onset of symptoms; the weak and inconsistent results are evidence against such a hypothesis. No application of the Pike and Smith method to HD has been reported.

Chen *et al.* (1987) have recently demonstrated in a simulation study that space–time interaction methods will lack statistical power against reasonable biological models for HD involving relatively low infectivity and long, variable latent periods.

Reasons for the use of these methods were three-fold: they were established methodology for infectious disease epidemiology, did not require detailed knowledge of the underlying population and were the only valid statistical approach to disease clustering.

### Social linkage studies

### *Methodology*

Vianna and Polan (1973) were the first to propose formal epidemiological designs for studying high-school contact amongst HD cases. In their 'two-time period' method high schools were classified according to the presence or absence of cases in two consecutive quinquennia and numbers of schools in the cells of a two-way table compared. The second 'index-secondary' method is a comparison of HD incidence rates in cohorts of high-school class-mates of cases (exposed cohorts) with those in similar unexposed cohorts. It is essential for both methods that case ascertainment be as complete as possible and particularly that it be free from geographic bias.

Case–control designs are available to study school, workplace or more general social linkage. Controls are typically matched to cases by date of birth, sex and area of residence and then linkage amongst case–case and control–control pairs compared (Pike & Smith, 1974). Statistical testing involves computer simulation (Zack *et al.*, 1977), permutation tests (Greenwald *et al.*, 1979) or direct derivation of the moments (Pike & Smith, 1974). Lack of ascertainment, if geographically unbiased, is less critical for these designs and would normally have a conservative effect. Eligibility criteria and matching rules are particularly important.

### *Applications*

Vianna and Polan (1973) identified eight high schools in Nassau and Suffolk counties with HD cases in 1960–64; of these five had cases in the next quinquennia compared with only three of the remaining 143 schools and with 0 of 16 matched control schools. This striking result represented a relative risk of 15 or more and raised considerable public concern. In the same study the index-secondary method yielded 28 observed cases and 10.2 expected. Extensive scrutiny of the methodology and numerous attempts at replication of the results have followed. Pike *et al.* (1974) criticised the results because of (possibly major) non-ascertainment of cases, which may be geographically biased (Grufferman & Delziel, 1984). Subsequent studies using these designs have yielded weakly positive results (Zack *et al.*, 1977: index-secondary method) or negative results (Zack *et al.*, 1977: two period method; Grufferman *et al.*, 1979; Paffenberger *et al.*, 1977). Of these, the only positive result has also been criticised for its exclusion of 17% of cases (Grufferman & Delziel, 1984). Thus these designs show fairly convincing evidence against a hypothesis involving transmission of an infective agent from HD cases when symptomatic.

The case–control studies, though usually interpreted as being equivalent, are sensitive to social linkage at quite different periods. There have been at least six studies (Zack *et al.*, 1977; Scherr *et al.*, 1984; Davis *et al.*, 1986; Smith *et al.*, 1977; Isador *et al.*, 1980; Davis, 1989) with analyses of HD cases alone as well as two including HD cases among others (Schimpff *et al.*, 1976; Greenwald *et al.*, 1979). Of the former, only two examined work-place linkage and found no evidence of increased risks. For school contact none suggest substantial risk but four of the six studies (all except Smith *et al.* and Isador *et al.*) report relative risks in the range 1.2–1.9. The studies differ in their use of geographic matching but this does not separate positive from negative studies. They also report different overall frequencies of school contact which depends on whether school or high-school attendence in the study area is an eligibility criterion. Contact at school is particularly low for the Oxford study (Smith *et al.*, 1977), suggesting that many subjects were educated outside the region. Since contact with other cases in the study will not be an accurate indicator of contact with other HD cases this is likely to yield a conservative bias. On the other hand, the positive results are of a magnitude which could be attributed to unexplained confounding by, for example, socioeconomic status.

Taken together the case–control studies are consistent with a hypothesis that some aspect of the shared social experience in school is a causative factor in the later development of HD. Whether primary schools (Davis, 1986, 1989) or high schools (most other studies) are most important remains obscure.

### Spatial clustering

### *Methodology*

Until recently applications of statistical methods for investigating spatial patterns to disease were hindered by two problems: the arbitrariness of the areas (census and other administrative units) for which denominator counts were available, and the marked heterogeneity of the distribution of the population at risk. In the past decade there has been considerable methodological interest in disease clustering with several new methods developed (e.g. Besag, 1989; Alexander *et al.*, 1989; Cuzick & Edwards, 1990; COMARE, 1989). Most of these analyse the distances between pairs of cases and especially to near neighbours after making appropriate adjustments for variation in the underlying population. Thus the NNA test (Alexander *et al.*, 1989; see also Besag, 1989) inspects each case in turn and determines whether it is unusually close to its near neighbours. In this event it is said to be a 'clustered case'. For the particular definitions used, 8% of all cases would be clustered if the distribution were purely random. Monte-Carlo methods test for significance proportions in excess of this 8%. As with spacio-temporal clustering there is little theoretical guidance on the comparative performance of the tests but a simulation study (Cartwright *et al.*, 1990) has demonstrated high statistical power for the NNA test against alternatives with 15–20% of all cases located as 'daughter' cases in small groups around 'parent' locations. Urquhart's method is not based on distances but compares the distribution of case counts in population units of approximately equal size made up by aggregating censal enumeration districts and has successfully identified clustering of meningococcal meningitis in Scotland.

In view of the results of Chen *et al.* (1987), discussed earlier, these statistical developments, the current availability of good quality small area population counts and the lack of precise biological models it is currently clear that spatial methods are preferable for analysing HD clustering.

### *Applications*

Abramson *et al.* (1980) reported spatial 'clustering' of HD in Israel but this was a tendency for cases to present in certain administrative regions and is not true clustering. The first report of an application of one of the new methods to HD (Alexander *et al.*, 1989) found statistically significant evidence of localised spatial clustering from the NNA test and the Cuzick–Edwards test for cases aged 0–34 at diagnosis, though not for older cases. In a companion paper (McKinney *et al.*, 1989) counts of cases by electoral wards had been examined and found to be non-random, again for younger cases. Shortly afterwards, Urquhart *et al.* (1989) reported similar results using their 'equal population method' for Scotland. These two series use extensively validated data from the Scottish cancer registries and from a specialist leukaemia/lymphoma registry (the Leukaemia Research Fund Data Collection Survey – DCS) covering approximately half of England and Wales. Cases registered by the DCS are collected according to a uniform protocol which aims to provide optimal ascertainment and to avoid geographical bias. The first results of the DCS analysis covered the period 1984–86 but have recently been confirmed over an extended series of 1,800 cases diagnosed in 1984–88 (Alexander *et al.*, submitted). A series of 741 white HD cases diagnosed in San Francisco has been analysed in a similar way (Glaser, 1989) with evidence of spatial clustering found in both young and older adults (15–34, ⩾55).

These analyses take location as residence at diagnosis. The results would be consistent with a relatively weak 'exposure' occurring within a few years of diagnosis or with a stronger effect associated with some earlier time period whose influence in the analyses was diluted by later migration. Alternative locations are possible and one report (Ross & Davis, 1989) finds clustering for childhood and teenage place of residence evident in cases diagnosed as young adults.

### Conclusions

The application of spatial clustering analyses to Hodgkin's disease is new and providing, at present, impressive consistency of results. The existence of spatial clustering at place of diagnosis for young adults is now a feature of the disease which aetiologic hypotheses must encompass.

Present results suggest that only a minority of such cases are linked by residential proximity at that time. This does not necessarily imply an absence of social linkage for the remainder but the rarity of HD spouse pairs, the negative results from the two case–control studies of work-place linkage and the equivocal results of space–time interaction tests would suggest otherwise. At the moment the balance of evidence leads to a concentration on events some time before diagnosis. In this case the spatial clustering takes closeness of location at diagnosis as a proxy for some (undetermined) linkage at an earlier period. That it has been consistently demonstrated so far suggests that this shared exposure must be associated with substantial excess risk.

The data are *consistent with* a hypothesis of personal transmission of a relatively rare infectious agent, possibly involving a small pool of carriers and/or long, close contact. They do not, however, *necessarily* bear this interpretation. They may also be interpreted in terms of shared participation in a social environment in which the infectious agent in a late-host-response model was epidemic rather than endemic. Alternatively there may be modulation of host response because 'herd immunity' is disregulated by population changes. A hypothesis of this sort has been suggested by Kinlen (1988, 1989) for childhood leukaemia and would be appropriate to the community experience described by Abramson (1980) and Vianna and Polan (1973). Animal models serve to emphasise that host response to potentially oncogenic viruses is crucially dependent on the social environment (Onions, 1987).

However, the spatial clustering may relate to some other aspect of the population or its common exposure acting synergistically with an infectious agent or independently (Grufferman *et al.*, 1977). Possibilities include genetic predisposation to viral secretion (Honeyman & Menser, 1974) or infection (McDevitt & Bodmer, 1974) and external environmental pollution (Plouffe, 1979). In this case the rationale for studying clustering is that the aetiologic agent may cluster in the same locations (Rothman, 1987).

The early studies of spatial clustering have mainly used routine registry data and consequently location at diagnosis. Further studies are essential, replicating these and in addition exploring locations at different times, especially during childhood and adolescence. Both types of study require complete ascertainment to maximise their statistical power as well as geographically uniform ascertainment to avoid bias. The second type may well form part of a new generation of case–control studies whose design will require careful consideration.

### References

ABRAMSON, J.H., GOLDBLUM, N., AVITZUR, M., PRIDAN, H., SACKS, M.I. & PERITZ, E. (1980). Clustering of Hodgkin's disease in Israel: a case–control study. *Int. J. Epidemiol.*, **2**, 137.

ALDERSON, M.R. & WAYAK, R. (1972). Epidemiology of Hodgkin's disease. *J. Chron. Dis.*, **25**, 253.

ALDERSON, M.R. & MAYAK, R. (1971). A study of space-time clustering of Hodgkin's disease in the Manchester region. *Br. J. Prev. Soc. Med.*, **25**, 168.

ALEXANDER, F.E., WILLIAMS, J., MCKINNEY, P.A., RICKETTS, T.J. & CARTWRIGHT, R.A. (1989). A specialist leukaemia/lymphoma registry in the UK. Part 2: clustering of Hodgkin's disease. *Br. J. Cancer*, **60**, 948.

BARTON, D.E., DAVID, F.N. & FIX, E. (1966). *Tests for Space–time Interaction and a Power Function*. Proceedings of the 5th Berkeley symposium on mathematical statistics and probability. University of California Press: Berkeley.

BESAG, J. (1989). Contribution to the discussion. RSS meeting on leukaemia clustering. *JRSS Series A*, **152**, 367.

CARTWRIGHT, R.A., ALEXANDER, F.E., MCKINNEY, P.A., RICKETTS, T.J., CLAYTON, D.G.C. & HAYHOE, F.G.H. (1990). *Leukaemia and Lymphoma*. An atlas of distribution 1984–1988 in parts of England and Wales. Leukaemia Research Fund: London.

CHEN, R., MANTEL, N. & KLINGBERG, M.A. (1984). A study of three techniques for time–space clustering in Hodgkin's disease. *Stat. Med.*, **3**, 173.

CLEMMENSON, J. (1981). To the epidemiology of Hodgkin's lymphogranulomatosis. *J. Belge Radiol.*, **3**, 263.

COMARE (Committee on Medical Aspects of Radiation in the Environment) (1988). Investigation of the possible increased incidence of leukaemia in young persons near the Dounreay Nuclear Establishment, Caithness, Scotland. OPCS: London.

CORREA, P. & O'CONOR, G.T. (1971). Epidemiologic patterns of Hodgkin's disease. *Int. J. Cancer*, **8**, 192.

CUZICK, J. & EDWARDS, R. (1990). Tests for spatial clustering of events in inhomogeneous populations. *J. R. Stat. Soc. Series B* (in the press).

DALGLEISH, A.G. & MCELWAIN, T. (1980). A viral aetiology for Hodgkin's disease. *Aust. NZ J. Med.*, **16**, 823.

DAVIS, S. (1986). Case aggregation in young adult Hodgkin's disease. Etiologic evidence from a population experience. *Cancer*, **51**, 1602.

DAVIS, S. (1989). Aggregation of Hodgkin's disease in school and employment settings. In *Abstracts of Scientific Presentations: National Conference on Clustering of Health Events*, p. 8. US Center for Disease Control: Atlanta, GA.

EDERER, F., MYERS, M.H. & MANTEL, N. (1964). A statistical problem in space and time: do leukaemia cases come in clusters? *Biometrics*, **20**, 626.

EVANS, A.R., HANCOCK, B.W., BROWN, M.J. & RICHMOND, J. (1977). A small cluster of Hodgkin's disease. *Br. Med. J.*, **i**, 1056.

EVANS, A.S. & GUTENSOHN, N.M. (1984). A population based case-control study of EBV and other viral anti-bodies among persons with Hodgkin's disease and their siblings. *Int. J. Cancer*, **34**, 149.

EVANS, A. & MUELLER, N. (1987). Prospective epidemiological studies of Epstein-Barr Virus in Hodgkin's disease in sera obtained prior to diagnosis. XI Scientific Meeting of the International Epidemiological Association, Helsinki.

FRAUMENI, J.F. & LI, F.P. (1969). Hodgkin's disease in childhood: an epidemiologic study. *J. Natl Cancer Inst.*, **42**, 681.

GLASER, S.L. (1989). Evidence for spatial clustering of Hodgkin's disease in the San Francisco Bay area. In *Abstracts of Scientific Presentations: National Conference on Clustering of Health Events*, p. 28. US Center for Disease Control: Atlanta, GA.

GREENBERG, R., GRUFFERMAN, S. & COLE, P. (1983). An evaluation of space-time clustering in Hodgkin's disease. *J. Chron. Dis.*, **36**, 257.

GREENWALD, P., ROSE, J.S. & DAITCH, P.B. (1979). Acquaintance networks among leukaemia and lymphoma patients. *Am. J. Epidemiol.*, **110**, 162.

GRUFFERMAN, S., COLE, P., SMITH, P.G. & LUKES, R.J. (1977). Hodgkin's disease in siblings. *N. Engl. J. Med.*, **296**, 248.

GRUFFERMAN, S., COLE, P. & LEVITAN, T. (1979). Evidence against transmission of Hodgkin's disease in high schools. *N. Engl. J. Med.*, **300**, 1006.

GRUFFERMAN, S. & DELZELL, E. (1984). *Epidemiology of Hodgkin's Disease*. Epidemiologic reviews, Johns Hopkins University.

GUTENSOHN, N. & COLE, P. (1977). Epidemiology of Hodgkin's disease in the young. *Int. J. Cancer*, **19**, 595.

GUTENSOHN, N. & COLE, P. (1980). Epidemiology of Hodgkin's disease. *Semin. Oncol.*, **7**, 92.

HEATH, C.W., EVERETT, J.R., STEWART, J.R., DAINES, J. & DAINES, P.H. (1973). Clustering in Hodgkin's disease. *Lancet*, **i**, 669.

HONEYMAN, M.C. & MENSER, M.A. (1974). Ethnicity is a significant factor in the epidemiology of rubella and Hodgkin's disease. *Nature*, **251**, 441.

ISAGAR, H. & LARSEN, S. (1980). Pre-morbid factors in Hodgkin's disease III. School contact among patients. *Scand. J. Haematol.*, **25**, 158.

KALIDI, I., MASSET, M., GONY, J. & 7 others (1989). MHC related genetic susceptibility to Hodgkin's disease. *Nouv. Rev. Fr. Hematol.*, **64**, 149.

KINLEN, L.J. (1988). Evidence for an infective cause of childhood leukaemia: comparison of a Scottish new town with nuclear reprocessing sites in Britain. *Lancet*, **ii**, 1323.

KINLEN, L.J. (1989). The relevance of population mixing to the aetiology of childhood leukaemia. In *Medical Response to Effects of Ionising Radiation*, Crosbie, W.A. & Gittus, J.H. (eds), p. 272. Elsevier: London.

KLINGER, R.J. & MINTON, J.P. (1973). Case clustering of Hodgkin's disease in a small rural community with association among cases. *Lancet*, **i**, 168.

KNOX, E.G. (1964). The detection of space-time interactions. *Appl. Stat.*, **13**, 25.

KRYSCIO, R.J., MYERS, M.H., PRUSINER, S.T., HEISE, H.W. & CHRISTINE, B.W. (1973). The space-time distribution of Hodgkin's disease in Connecticut 1940–1969. *J. Natl Cancer Inst.*, **50**, 1107.

MCDEVITT, H.O. & BODMER, W.F. (1974). HLA Immune response genes and disease. *Lancet*, **ii**, 1269.

MCKINNEY, P.A., ALEXANDER, F.E., RICKETTS, T.J., WILLIAMS, J. & CARTWRIGHT, R.A. (1989). A specialist leukaemia/lymphoma registry in the UK. Part 1: incidence and geographical distribution of Hodgkin's disease. *Br. J. Cancer*, **60**, 942.

MCMAHON, B. (1966). Epidemiology of Hodgkin's disease. *Cancer Res.*, **26**, 1189.

MANGOUD, A., HILLIER, V.F., LECK, I. & THOMAS, R.W. (1985). Space-time interaction in Hodgkin's disease in Greater Manchester. *J. Epidemiol. Comm. Hlth*, **39**, 58.

MUNOZ, N., DAVIDSON, R.L.J., WITTHOFF, B., ERICSSON, J.E. & DE-THE, G. (1978). Infectious mononucleosis and Hodgkin's disease. *Int. J. Cancer*, **22**, 10.

ONIONS, D. (1987). *Epidemiology of Feline Leukaemia Virus Infections*. Balliere: London.

PAFFENBERGER, R.S., WING, A.L. & HYDE, R.T. (1977). Characteristics in youth indicative of adult-onset Hodgkin's disease. *J. Natl Cancer Inst.*, **58**, 1489.

PIKE, M.C., HENDERSON, B.E., CASAGRANDE, J., SMITH, P.G. & KINLEN, L.J. (1974). Infectious aspects of Hodgkin's disease. *N. Engl. J. Med.*, 290.

PIKE, M.C. & SMITH, P.G. (1974). Clustering of cases of Hodgkin's disease and leukaemia. *Cancer*, **34**, 1390.

PIKE, M.C. & SMITH, P.G. (1968). Disease clustering: a generalisation of Knox's approach to the detection of space-time interactions. *Biometrics*, **24**, 541.

PINKEL, D., DOWD, J.E. & BROSS, I.D.J. (1963). Some epidemiological features of malignant solid tumours of children in the Buffalo, NY area. *Cancer*, **16**, 28.

PLOUFFE, J.F., SILVA, J., SCHWARTZ, R.S. & 5 others (1979). Abnormal lymphocyte responses in residents of a town with a cluster of Hodgkin's disease. *Clin. Exp. Immunol.*, **35**, 163.

RASIZ, D.V., DIAMOND, H.D. & CRAVER, L.F. (1959). Familial Hodgkin's disease: its significance and implications. *Ann. Intern. Med.*, **51**, 933.

ROSS, A. & DAVIS, S. (1989). Point pattern analysis of Hodgkin's disease residences prior to diagnosis. In *Abstracts of Scientific Presentations: National Conference on Clustering of Health Events*, p. 6. US Center for Disease Control: Atlanta, GA.

ROTHMAN, K.J. (1987). Clustering of disease (editorial). *Am. J. Pub. Hlth*, **78**, 306.

SCHERR, P.A., GUTENSOHN, N. & COLE, P. (1984). School contact among persons with Hodgkin's disease. *Am. J. Epidemiol.*, **120**, 29.

SCHIMPFF, S.C., BRAGER, D.M., SCHIMPFF, C.R., COMSTOCK, G.W. & WIERNIK, P.H. (1976). Leukaemia and lymphoma patients linked by prior social contact. Evaluation using a case-control approach. *Ann. Intern. Med.*, **84**, 547.

SCHWARTZ, R.S., CALLEN, J.P. & SILVA, J. (1978). A cluster of Hodgkin's disease in a small community. Evidence for environmental factors. *Am. J. Epidemiol.*, **108**, 19.

SMITH, P.G. & PIKE, M.C. (1976). Current epidemiological evidence for transmission of Hodgkin's disease. *Cancer Res.*, **36**, 660.

SMITH, P.G., PIKE, M.C., KINLEN, L.J. & JONES, A. (1977). Contacts between young patients with Hodgkin's disease. *Lancet*, **ii**, 59.

SMITH, P.G. (1982). Spatial and temporal clustering. In *Cancer Epidemiology and Prevention*, Schottenfeld, D. & Fraumeni, J.F. (eds), p. 391. Sanderson: Philadelphia.

STAAL, S.P., ABINDER, R., BESCHORNER, W.E., HAYWARD, G.S. & MANN, R. (1989). A survey of Epstein-Barr Virus DNA in lymphoid tissue. *Am. J. Clin. Pathol.*, **91**, 1.

URQUHURT, J., BLACK, R. & BUIST, E. (1989). Exploring small area methods. In *Methodology of Enquiries into Disease Clustering*. SAHSU: London.

VIANNA, N.J., GREENWALD, P. & DAVIES, J.N.P. (1971). Extended epidemic of Hodgkin's disease in high-school students. *Lancet*, **i**, 1209.

VIANNA, N.J., GREENWALD, P., BRADY, J. & 4 others (1972). Hodgkin's disease: cases with features of a community outbreak. *Ann. Intern. Med.*, **77**, 169.

VIANNA, N.J. & POLAN, A.K. (1973). Epidemiologic evidence for transmission of Hodgkin's disease. *N. Engl. J. Med.*, **289**, 499.

VIANNA, N.J., DAVIES, J.N.P., POLAN, A.K. & WOLFGANG, P. (1974). Familial Hodgkin's disease: an environmental and genetic disorder. *Lancet*, **ii**, 854.

WEISS, L.M., STRICKLER, J.G., WARNKE, R.A., PURTILO, D.T. & SKLER, J. (1987). Epstein-Bar Viral DNA in tissues of Hodgkin's disease. *Am. J. Pathol.*, **129**, 86.

WEISS, L.M., MOVAHED, L., WARNKE, R.A. & SKLAR, J. (1989). Detection of Epstein-Barr viral genomes in Reed Sternberg cells of Hodgkin's disease. *N. Engl. J. Med.*, **320**, 502.

ZACK, M.W., HEATH, C.W., ANDREWS, M. DE W., GRIVAS, A.S. & CHRISTINE, B.W. (1977). High school contact among persons with leukaemia and lymphoma. *J. Natl Cancer Inst.*, **59**, 1343.